# Prediction model of all-cause death based on balance ability among middle-aged and older Chinese adults of overweight and obesity

**DOI:** 10.3389/fpubh.2022.1039718

**Published:** 2022-12-22

**Authors:** Kaihong Xie, Xiao Han, Jia Lu, Xiao Xu, Xuanhan Hu

**Affiliations:** ^1^School of Nursing, Zhejiang Chinese Medical University, Hangzhou, China; ^2^School of Health Humanities, Peking University Health Science Center, Beijing, China; ^3^The Second School of Clinical, Zhejiang Chinese Medical University, Hangzhou, China

**Keywords:** balance ability, death, nomogram, prediction model, CHARLS

## Abstract

**Background:**

Advances in studies using body indicators to predict death risk. Estimating the balance ability of death risk in middle-aged and older Chinese adults with overweight and obesity is still challenging.

**Methods:**

A retrospective analysis of the data from the China Health and Retirement Study from January 2011 to December 2018. A total of 8,632 participants were randomly divided into 7:3 a training group and a verification group, respectively. Univariable Cox analysis was used to prescreen 17 potential predictors for incorporation in the subsequent multivariable Cox analysis. Nine variables were included in the nomogram finally and validated with concordance index (C-index), calibration plots, Hosmer-Lemeshow test, and internal validation population.

**Results:**

287 participants were death in the training group. One hundred and thirteen participants were death in the verification group. A total of nine indicators were included in the modeling group, including gender, age, marriage, hypertension, diabetes, stroke, ADL, IADL, and balance ability to establish a prediction model. The nomogram predicted death with a validated concordance index of (C-index = 0.77, 95% CI: 0.74–0.80). The inclusion of balance ability variables in the nomogram maintained predictive accuracy (C-index = 0.77, 95% CI: 0.73–0.82). The calibration curve graph and Hosmer-Lemeshow test (*P* > 0.05 for both the modeling group and the verification group) showed the model has a good model consistency.

**Conclusion:**

In the present study, we provide a basis for developing a prediction model for middle-aged and older people with overweight and obesity. In most cases, balance ability is more reversible than other predictors.

## 1. Introduction

Balance ability is the basis of the movement toward the human body. It was divided into static balance and dynamic balance. Static balance refers to the ability of the body or a certain part of the body to remain stable when it is in a certain posture, such as sitting or standing. Balance ability depended on the mutual integration and coordination of the visual system, somatosensory system, and vestibular system (1). Impaired balance ability might lead to adverse outcomes such as decreased walking ability, impaired cardiopulmonary function, and accidental falls (2). Balance ability test can be a part of routine physical examination in middle-aged and older adults (3). Studies have shown participants with poor balance ability were at higher risk of cognitive impairment (4). Impairment of balance ability not only caused panic in middle-aged and older adults (5), but also impaired their physical functions (6). It also increased their families' socioeconomic burdens (7).

The physical function impairment degree was highly correlated with hazard event severity and the individual's physiologic reserve (8). Studies have confirmed that physical performance measures, including walking speed and balance, were useful predictors of adverse health outcomes (9). Multiple body indicators (including balance) can predict death and improve the performance of prediction models (10). Previous studies showed age and diabetes were indicators for death (11, 12). The Short Physical Performance Battery (SPPB) (13) is one of the most effective tools for assessing this, but how balance ability indicates all-cause death in middle-aged and old Chinese adults with overweight and obesity has not been studied. The current research focused on the combination of multiple body indicators as a basis of clinical prediction of death. Cox proportional hazard models or areas under ROC curve (a tool to test the desirable accuracy and sensitivity) (14) were used to assess the risk. We lack the predictive model. We tend to use the ability of balance to predict death.

We posed the hypothesis that balance ability is associated with all-cause death in middle-aged and older adults with overweight and obesity and we can predict all-cause death.

## 2. Manuscript formatting

### 2.1. Ethics statement

The Ethical Review Committee at Peking University (IRB 00001052–11014) approved CHARLS for the biomarker sample collection.

### 2.2. Study population

**Figure 1:**
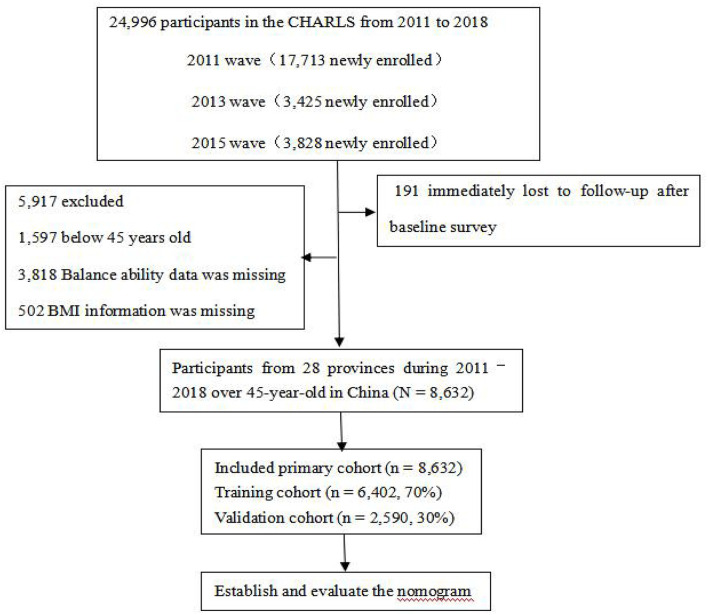
Flowchart of sample selection.

The population used in the present study consisted of adults 45 years of age and older in the China Health and Retirement Longitudinal Study (CHARLS), which included 24,805 participants in 2011–2018 and had national representation in China. All respondents completed a structured questionnaire on sociodemographic features, lifestyles, and health-related behaviors/conditions. Participants signed formal informed consent. We excluded participants below 45-year-old for all analyses *(n* = 1,597). Participants who did not test balance ability (*n* = 3,818) were excluded, resulting in a final sample size of 19,390. Additionally, participants did not have BMI measures were excluded (*n* = 502). We included the participants with overweight and obesity for a sample size of 8,632. Finally, 6,042 participants in 2011–2018 were included into the training cohort, while 2,590 were included into the verification cohort (Figure 1). As for longitudinal follow-up, participants were followed for 1-, 5-, and 7- years, respectively.

### 2.3. Main indicator

The participants were asked to maintain their balance for the full 10 s during the balance ability test. The maximum value is 10 s. The participant was instructed to stand with the side of the heel of one foot touching the big toe of the other foot for 10 s, if they were able. The respondent is allowed to put either foot in front and may use their arms, bend their knees or use their body to maintain balance. Balance ability was coded as 10 s if the respondent maintained their balance for 10 s, while an exact number of seconds indicates that the respondent's time fell below 10 s. We generated binary variables based on the median of balance ability. Codes were missing if participants refused to complete the test for safety reasons or for other reasons.

### 2.4. Other indicators

The participants were asked about their exact age, gender (male/female), marriage, and health status. Marriage was divided into married and non-married. Whether the participants were diagnosed with a disease (hypertension, diabetes, and stroke) was classified as Yes or No. Participants with or without activities of daily living/instrumental activities of daily living (ADLs/IADLs) were also divided into Yes and No, Yes means limited by more than one or not. According to our conceptual framework, low socioeconomic position and healthy status is a strong determinant of all-cause death. Socio-demographic status included *hukou*, income, and education. Education was classified into five categories (no formal education, primary school, middle or high school, and college or above). *hukou* (15) (household register in China) included agricultural *hukou* and non-agricultural *hukou*. Health-related factors were self-reported smoking, drinking alcohol (never, former, or current), diagnosed diseases (chronic lung diseases and memory diseases), fall, hip fraction, and body mass index (BMI).

### 2.5. Outcome

The death information for each participant, the outcome variable, was obtained from death registration and certification by asking their relatives or community managers in 2013, 2015, and 2018, or at the end of the follow-up (March 31, 2019). Death events are identified as “1,” survival and censorship “0.” The survival time is calculated according to the outcome (censored participations' survey year counted as survival time).

### 2.6. Statistical analysis

Data were analyzed using the statistical software SAS (version 9.4) and R (version 4.2.1). For summary statistics, we employed means and standard deviation (*SD*s) to describe continuous variables (normal distribution). Categorical variables were described by frequency and percentage. We used the χ^2^-test, analysis of variance, and Mann-Whitney *U*-test to determine the significance of inter-group differences. We randomly assigned 70% of the patients to the training cohort (*n* = 6,042) and 30% of the patients to the validation cohort (*n* = 2,590). We used the training cohort to evaluate the effect of the unknown models and estimated the performance of the model unbiasedly. We validated the known models and select the optimal model with a validation cohort. Assumed to be missing at random, missing values were imputed with multivariate imputation of classification or regression trees [missing data 8.1% (702/8,632)]. Ten imputed data sets were created and pooled into R software. *P* < 0.05 was considered statistically significant.

Univariate Cox proportional hazard regression was performed to evaluate each statistically significant variable in the training cohort. The variables with *P* < 0.05 were included in a final multivariate Cox proportional hazard regression using the forward step-up selection procedure, with a liberal *P* < 0.05 as the retention criteria to select the independent risk factors for all-cause death of the middle-aged and older adults with overweight and obesity. The proportional hazards assumption was justified for the participants (*P* = 0.760). Cox proportional hazard regression models were performed to examine the hazard of balance ability exposure measure and all-cause death. Model 1 adjusted for gender and age. Model 2 was further adjusted for marital status. Model 3 was Model 2 plus hypertension, diabetes, stroke, and ADL/IADL. Survival curves were estimated by Kaplan-Meier analysis in different balance ability in model 3. All these analyses were evaluated by original data to test the sensitivity. The estimate of relative risk was evaluated by the Hazard ratio (HR) with a 95% confidence interval (CI).

Finally, a nomogram was built based on the result of multivariate Cox, and the “rms” (version 5.1–4) package was used for creating the nomogram. Verification and evaluation of the nomogram. Verifying and evaluating the performance of a nomogram involves the use of verification sets for identification and calibration. The consistency between the predicted probabilities and actual results was evaluated in plots. The relative corrected consistency index (C-index) of the nomogram was also determined. The C-index quantified the predictive ability of the model. The perfect prediction should fall on a 45° straight line passing through the origin. We performed bootstrapping with 1,000 resamples.

## 3. Results

### 3.1. Patient characteristics

A total of 8,632 eligible middle-aged and older adults with overweight and obesity were involved in the present study, including 3,746 men (43.4%) and 4,881 women (56.5%), with 56.3 ± 8.8 years old. The overall all-cause death was 4.63% (*n* = 400). Among them, 8,632 patients were included in the training cohort, and 6,042 patients were included in the validation cohort (Table 1). Participants who failed the balance test were slightly older and more likely to be female, have less education, be in Agricultural *hukou*, be diabetic, stroke and hypertensive, suffer from memory diseases, fall, hip fraction, and with ADL/IADL.

**Table 1:** Comparison between training and validation cohorts.

**Characteristics**	**Labels**	**Training cohort (*n* = 6,042)**		**Validation cohort (*n* = 2,590)**		***P***
		**Subjects with**	**Subjects with**	**Subjects with**	**Subjects with**	
		**failed tandem**	**completed**	**failed tandem**	**completed**	
		**stance (*****n*** = **32)**	**tandem stance**	**stance (*****n*** = **20)**	**tandem stance**	
			**(*****n*** = **6,010)**		**(*****n*** = **2,570)**	
Age, mean (SD)		63.69 (8.7)	56.20 (8.8)	66.20 (11.3)	56.36 (8.7)	0.334
Gender (%)	Male	8 (25.0)	2,582 (43.0)	2 (10.0)	1,154 (44.9)	0.138
	Female	24 (75.0)	3,424 (57.0)	18 (90.0)	1,415 (55.1)	
Marriage status (%)						
	Yes	29 (90.6)	5,453 (90.7)	17 (85.0)	2,355 (91.6)	0.221
	No	3 (9.4)	557 (9.3)	3 (15.0)	215 (8.4)	
*hukou* (%)						
	Agricultural	24 (77.4)	4,050 (73.2)	16 (80.0)	1,719 (72.5)	0.563
	Non-agricultural	7 (22.6)	1,483 (26.8)	4 (20.0)	652 (27.5)	
Education (%)						
	No formal education	23 (71.9)	2,187 (39.3)	14 (70.0)	921 (38.6)	0.897
	Primary school	7 (21.9)	1,206 (21.7)	4 (20.0)	518 (21.7)	
	Middle or high school	2 (6.2)	2,009 (36.1)	2 (10.0)	881 (36.9)	
	College or above	0 (0.0)	165 (3.0)	0 (0.0)	67 (2.8)	
Smoking status (%)						
	Never	4 (12.5)	1,487 (24.8)	1 (5.0)	654 (25.5)	0.164
	Formal	3 (9.4)	512 (8.5)	3 (15.0)	246 (9.6)	
	Current	25 (78.1)	4,002 (66.7)	16 (80.0)	1,663 (64.9)	
Drinking status (%)						
	Never	5 (15.6)	1,412 (23.5)	1 (5.0)	641 (25.0)	0.281
	Formal	1 (3.1)	525 (8.8)	1 (5.0)	235 (9.2)	
	Current	26 (81.2)	4,063 (67.7)	18 (90.0)	1,689 (65.8)	
History of comorbidities (%)						
	Hypertension	12 (37.5)	1,713 (28.5)	8 (40.0)	723 (28.1)	0.778
	Diabetes	4 (12.5)	431 (7.2)	2 (10.0)	165 (6.4)	0.226
	Chronic lung diseases	2 (6.2)	448 (7.5)	1 (5.0)	205 (8.0)	0.442
	Stroke	2 (6.2)	117 (1.9)	1 (5.0)	35 (1.4)	0.077
	Memory diseases	1 (3.1)	58 (1.0)	0 (0.0)	27 (1.1)	0.869
ADL^a^ (%)	No	26 (81.2)	5,357 (89.1)	15 (75.0)	2,311 (89.9)	0.344
	Yes	6 (18.8)	653 (10.9)	5 (25.0)	259 (10.1)	
IADL^b^ (%)	No	25 (78.1)	5,097 (84.8)	10 (50.0)	2,230 (86.8)	0.043
	Yes	7 (21.9)	913 (15.2)	10 (50.0)	340 (13.2)	
Depression (%)	No	16 (50.0)	3,977 (66.2)	14 (70.0)	1,719 (66.9)	0.473
	Yes	16 (50.0)	2,033 (33.8)	6 (30.0)	851 (33.1)	
Fall (%)	Yes	9 (28.1)	897 (15.5)	7 (35.0)	373 (15.0)	0.637
	No	23 (71.9)	4,874 (84.5)	13 (65.0)	2,111 (85.0)	
Hip fraction (%)	Yes	0 (0.0)	79 (1.4)	0 (0.0)	35 (1.4)	0.978
	No	32 (100.0)	5,696 (98.6)	20 (100.0)	2,451 (98.6)	

SD, standard deviation.

^a^P-value was based on χ or analysis of variance or Mann-Whitney U-test whenever appropriate.

^b^Calculated as weighi͡n kilograms divided by height in meters squared.

### 3.2. Independent prognostic factors in the study population

**Figure 2:**
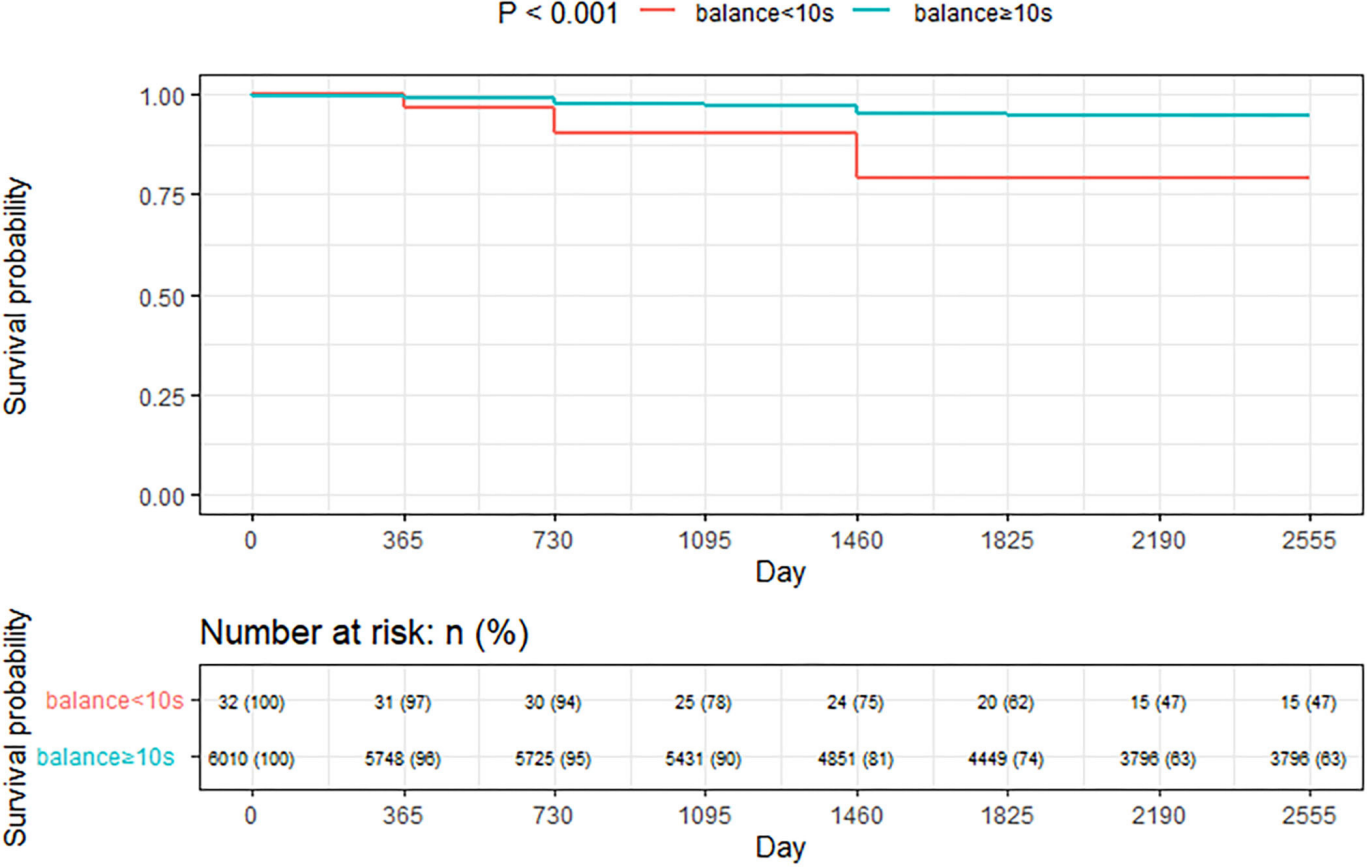
Kaplan-Meier survival for the stratified by balance ability.

Kaplan-Meier method was used to calculate the median survival time of handgrip strength variable on all-cause death (Figure 2). Nine variables were entered into the multivariate cox regression analysis of all-cause death with the estimate of collinearity. Age, gender, marriage status, hypertension, diabetes, stroke, ADL/IADL, and Balance ability were regarded as the independent prognostic factors of all-cause death (Table 2). In the longitudinal analyses, worse balance ability was associated with a high risk of all-cause death, and this association was present after the adjustment for potential confounders. Additionally, the Kaplan-Meier curves showed that much more all-cause deaths occurred at balance ability < 10 s when compared to balance ability ≥ 10 s (*p* < 0.001; Figure 2). We conducted a sensitivity analysis to demonstrate the positive effect of good balance ability to all-cause deaths (p < 0.05; Table 3).

**Table 2:** The independent risk factors of all-cause death in the multivariate Cox hazards model in development group.

	***B***	**HR**	**95% CI**	***P***
Age	0.07	1.07	1.06–1.09	< 0.001
Gender	−0.64	0.53	0.41–0.68	< 0.001
Marriage status	0.53	1.71	1.25–2.32	0.001
Hypertension	0.35	1.42	1.11–1.92	0.005
Diabetes	0.48	1.62	1.17–2.25	0.004
Stroke	0.50	1.66	1.01–2.72	0.047
ADL	0.48	1.61	1.19–2.19	0.002
IADL	0.33	1.39	1.04–1.86	0.028
Balance ability	−0.17	0.84	0.75–0.95	0.005

**Table 3:** Models for all-cause death according to balance ability.

**Groups**	**Number**	**Development cohort [HR (95% CI)]**			**Number**	**Validation cohort [HR (95% CI)]**		
		**Model 1**	**Model 2**	**Model 3**		**Model 1**	**Model 2**	**Model 3**
Balance ability	6,042	0.84 (0.74–0.94)^*^	0.83 (0.73–0.93)^*^	0.83 (0.74–0.94)^*^	2,590	0.84 (0.74–0.94)^*^	0.83 (0.73–0.93)^*^	0.83 (0.74–0.94)^*^
Balance ability								
< 10 s	32	1	1	1	20	1	1	1
≥10 s	6,010	0.36 (0.16–0.82)^*^	0.35 (0.15–0.78)^*^	0.35 (0.16–0.80)^*^	2,570	0.36 (0.16–0.82)^*^	0.35 (0.15–0.78)^*^	0.35 (0.16–0.80)^*^

Model 1 adjusted for gender and age. Model 2 further adjusted for marriage status. Model 3 was Model 2 plus hypertension, diabetes, stroke, and ADL/IADL.

^*^ means *P* < 0.05.

### 3.3. Nomogram development and validation

**Figure 3:**
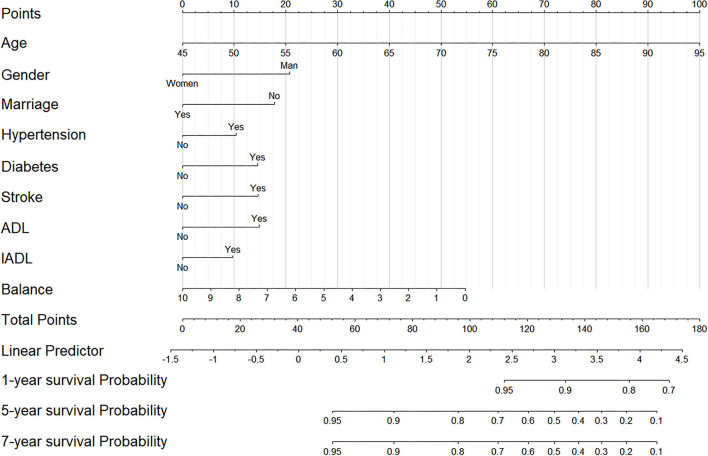
The nomogram predicting 1-, 5-, and 7-year cumulative probabilities of all-cause death in participants with overweight and obesity.

**Figure 4:**
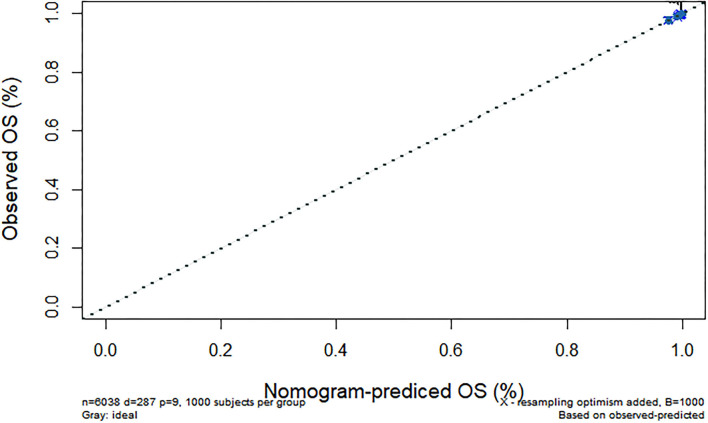
Predicted 1-year all-cause death probability (training group). Calibration curves of 1-, 5-, and 7-year all-cause death drop out. The x-axis is the predicted probability and the y-axis is the actual probability. Based on the prediction model, we predicted and analyzed calibration curves for 1-, 5-, and 7 years of all-cause death.

**Figure 5:**
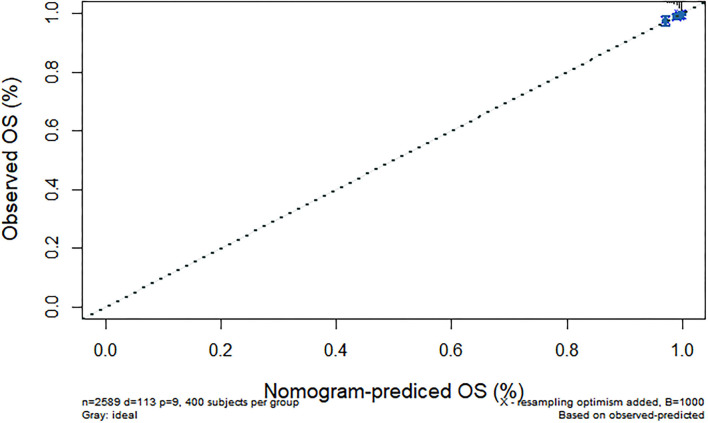
Predicted 1-year all-cause death probability (verification group). Calibration curves of 1-, 5-, and 7-year all-cause death drop out. The x-axis is the predicted probability and the y-axis is the actual probability. Based on the prediction model, we predicted and analyzed calibration curves for 1-, 5-, and 7 years of all-cause death.

**Figure 6:**
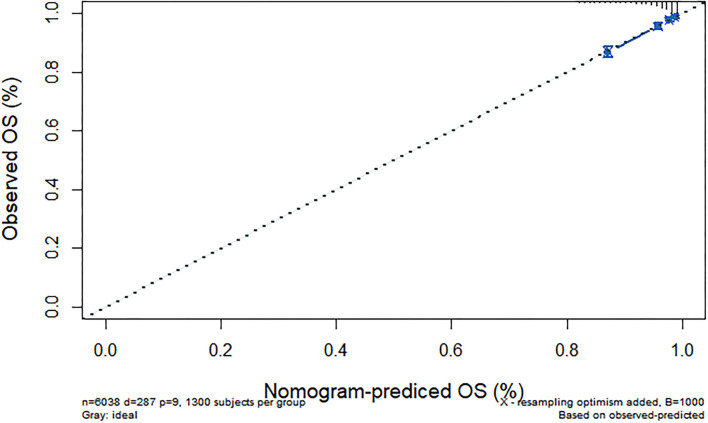
Predicted 5-year all-cause death probability (training group). Calibration curves of 1-, 5-, and 7-year all-cause death drop out. The x-axis is the predicted probability and the y-axis is the actual probability. Based on the prediction model, we predicted and analyzed calibration curves for 1-, 5-, and 7 years of all-cause death.

**Figure 7:**
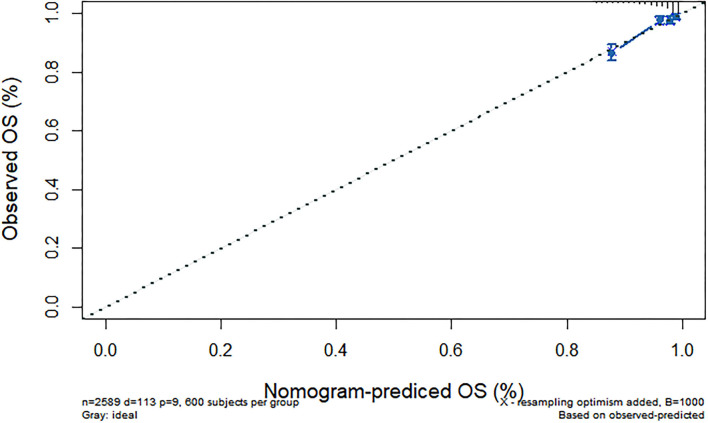
Predicted 5-year all-cause death probability (verification group). Calibration curves of 1-, 5-, and 7-year all-cause death drop out. The x-axis is the predicted probability and the y-axis is the actual probability. Based on the prediction model, we predicted and analyzed calibration curves for 1-, 5-, and 7 years of all-cause death.

**Figure 8:**
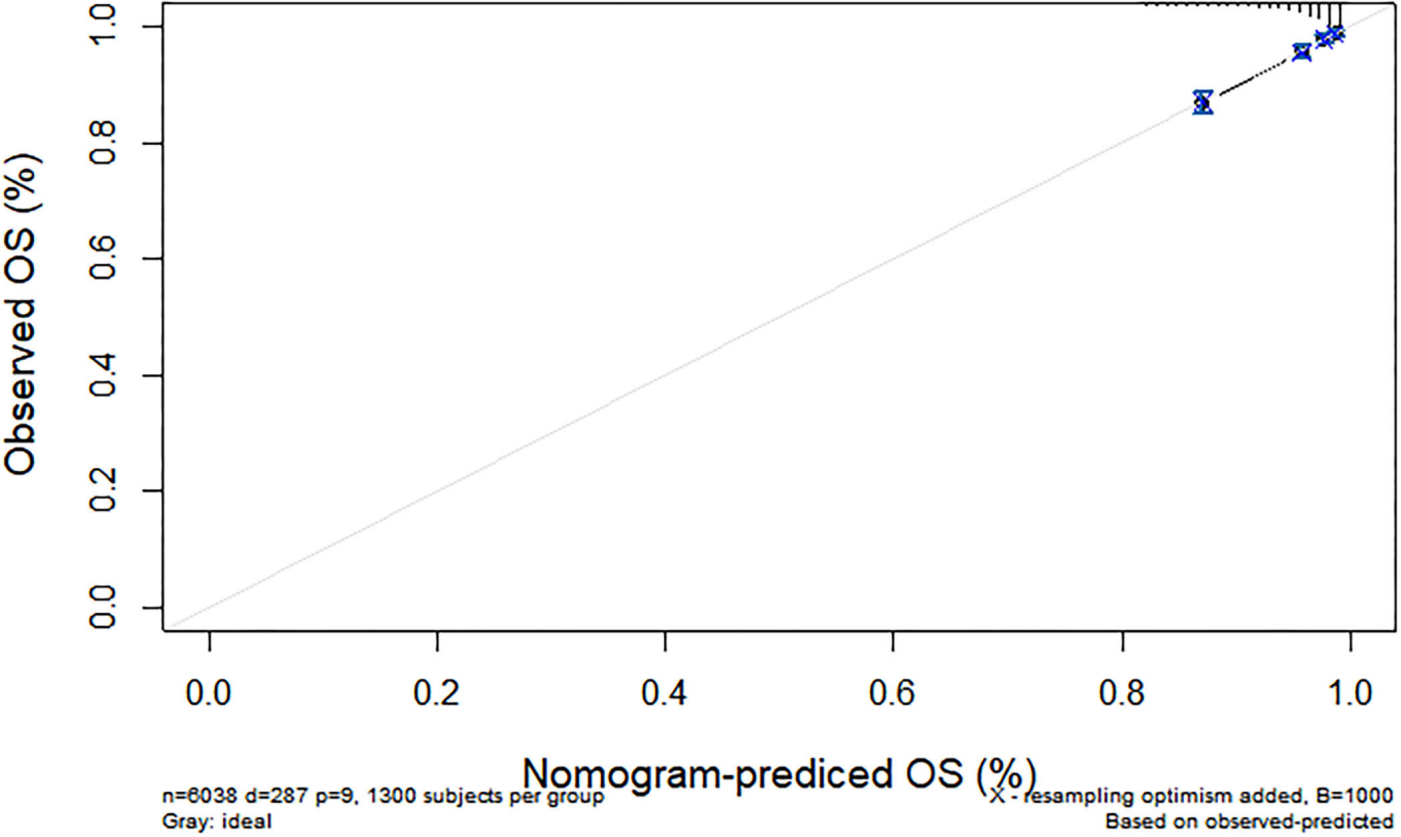
Predicted 7-year all-cause death probability (training group). Calibration curves of 1-, 5-, and 7-year all-cause death drop out. The x-axis is the predicted probability and the y-axis is the actual probability. Based on the prediction model, we predicted and analyzed calibration curves for 1-, 5-, and 7 years of all-cause death.

**Figure 9:**
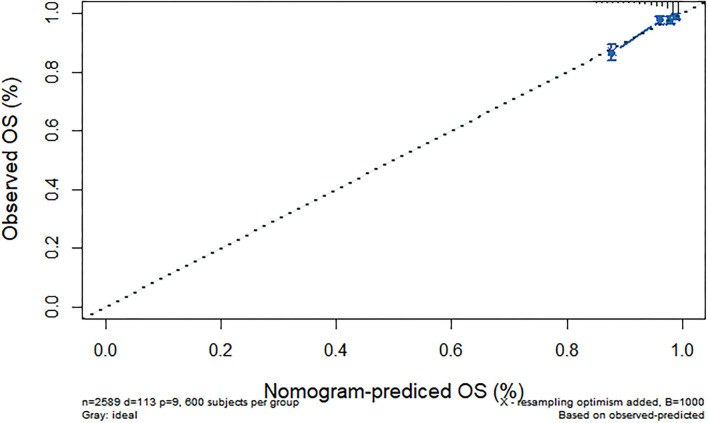
Predicted 7-year all-cause death probability (verification group). Calibration curves of 1-, 5-, and 7-year all-cause death drop out. The x-axis is the predicted probability and the y-axis is the actual probability. Based on the prediction model, we predicted and analyzed calibration curves for 1-, 5-, and 7 years of all-cause death.

We further constructed and predicted a nomogram for all-cause death, with the participant's age, gender, marriage, diabetes, hypertension, stroke, and ADL/IADL as relevant factors (Figure 3). Each prognostic factor was given a score on the point scale. Clinicians were able to estimate 1-, 5-, and 7-year survival rates among middle-aged and older Chinese adults with overweight and obesity by determining the score of each prognostic factor and calculating their total score. The C-index of this model was (C-index = 0.77, 95% CI: 0.74–0.80) in development group and (C-index = 0.77, 95% CI: 0.73–0.82) in validation group. Based on this prediction model, we predicted and analyzed calibration curves for 1, 5, and 7 years of all-cause death and found the model has a good model consistency (Figures 4–9).

## 4. Discussion

Our study focused on the 1-, 5-, and 7-year death risk prediction models for Chinese adults 45 years of age and older with overweight and obesity. The results were analyzed by C-index, calibration curve, and Hosmer-Lemeshow test. Studies have proved the feasibility and importance of nomograms in predicting all-cause deaths (16). In our study, a nomogram of all-cause mortality in Chinese adults 45 years of age and older with overweight and obesity was established based on the risk factors obtained by Cox regression analysis. The nomogram shows good predictive ability and clinical applicability.

Based on a large sample data from China, our study systematically assessed the mortality risk and influencing factors of Chinese adults 45 years of age and older with overweight and obesity, and based on this, predicted their survival probability. The survival probability of participants with high balance ability in the 3rd and 7th years is 12 and 16% higher than that participants with poor balance ability. Further research found that advanced age, male, non-married, hypertension, diabetes, stroke, and ADL/IADL dysfunction were risk factors for death. A good balance ability was the protective factor for death in Chinese 45 aged and over with overweight and obesity. On this basis, the death risk model established has a good prediction ability.

A good balance ability was a protective factor of all-cause death for Chinese adults 45 years of age and older with overweight and obesity. Middle-aged and older adults, especially those who were overweight and obese, manifest non-specific symptoms and signs, which usually included the decline in balance ability (17). Loss of muscle strength was an important factor in the balance dysfunction with age (18). Female muscle mass and strength declined faster than males (19), especially for middle-aged women in menopause (20), which was contrary to the fact that women are protective factors for the death risk of overweight and obesity among middle-aged and older people. Balance ability decreased with age. The older, the more severe consequences (21). The BMI also increases with age. Being overweight and obesity becomes one of the most important factors affecting balance (22), which caused lower foot sensitivity, poor balance and posture stability (23), increases the risk of imbalance (24), and lower limb injuries (25). Overweight and obesity also increased plantar shear stress (26). This is consistent with the study that standing balance can predict death (27). Physical performance proved a vital and early sign indicative of vulnerability that reflects several underlying physiological impairments. However, limited time and resources hinder the use of many available tools assessing physical performance in acute healthcare settings. Balance can be improved through training in a short time compared to age, gender, and chronic diseases (28). Our results indicate that balance ability, an easy-to-administer instrument can be a feasible method to assess physical performance in middle-aged and older participants.

At present, most studies only used Cox proportional hazard model or area under ROC curve to evaluate risk, lack of prediction model. Balance ability is a cheap and reversible factor. In middle-aged and older adults with overweight and obesity, self-balance ability test should be used to evaluate the patient's balance ability. Moreover, improving the balance of middle-aged and older adults with overweight and obesity through feasible interventions may ultimately reduce the risk of death through a complex set of mechanisms.

Age was a risk factor for death in participants with overweight and obesity. Peripheral sensations in middle-aged and older people were impaired to varying degrees with age, accompanied by decreased muscle mass and prolonged reaction time, aggravating isolation from the outside world (29). In addition to its own aging mechanism, BMI increased with age (30), often accompanied by a variety of complications, such as hypertension, cardiac insufficiency, which may accelerate the process of death (31). Female was a protective factor of death in middle-aged and older people with overweight and obesity. The gender factor may be related to genetic and environmental factors (32), male was more likely to smoke and drink than females. The incidence of fatal diseases in males was higher than that in females, such as cancer and cardiovascular diseases (33). Both at home and abroad, females were more sensitive to their own health evaluation. Women took medical treatment earlier and more frequent than men, which may be an important factor in early detection of death risk (34). Married status was a protective factor for all-cause death. Married participants received support from their spouses, such as medical assistance, assistance in daily activities and drug reminders (35). The divorced were vulnerable to alcohol and tobacco consumption, drug abuse and sexual problems (36). In addition, married people may have greater financial resources than unmarried people, which can detect early death risks and treat them in a timely manner (37).

Participants with hypertension, diabetes, or stroke were at risk factors for death. Brain injury such as stroke can lead to neuronal cell damage and death, which affects the motor and cognitive function of patients. Cardiac compensation reserves in patients with hypertension may be reduced, resulting in impaired adaptive response to acute circulatory stress. Increased capillary pressure may cause vascular rupture and bleeding, and damage to liver and kidney function. People with diabetes may experience systemic weakness, blurred vision and impaired lower limb function, resulting in loss of physical independence and accelerated muscle strength declined (38). All of these reasons may accelerate the death process.

Middle-aged and older adults with ADL or IADL dysfunction were at risk factors for death, which were consistent with the predicted results of two death risk models among the participants (39). The life expectancy of people with ADL disability was 2 years shorter than that of normal people, and people with ADL disability 85 years of age and older were more likely to die within 1 year. Although disability was a reversible process, some people cannot fully recover once they become disabled. A study showed that participants who died of diabetes experienced the highest level of ADL dysfunction 15 years before death. In the 5th year before death, all participants had physical disabilities. The participants who died of kidney disease, diabetes, stroke and hypertension reported at least one ADL dysfunction. ADL scores increased dramatically in subjects who died of stroke, diabetes and hypertension in the last year before death (40).

This study has limitations. We do not involve multiple causes of death, but the general concept of death from all causes. Participants, who were not measured to balance ability and with abnormal BMI were deleted, might have differences in baseline characteristics, which would affect the results. This study suggests that may be applicable to a wider or heterogeneous population, and further research is needed to confirm.

## 5. Conclusion

Balance ability was an economical and adjustable indicator of all-cause death in middle-aged and older Chinese adults with overweight and obesity. A nomogram was provided that incorporates a simple measure and basic information to calculate the risk of death. Simple indicators will facilitate clinical and rehabilitation work.

## Data availability statement

Publicly available datasets were analyzed in this study. This data can be found at: https://charls.pku.edu.cn/en/.

## Ethics statement

The studies involving human participants were reviewed and approved by Peking University (IRB 00001052–11014). The patients/participants provided their written informed consent to participate in this study. Written informed consent was obtained from the individual(s) for the publication of any potentially identifiable images or data included in this article.

## Author contributions

KHX planned the study, supervised the data analysis, and wrote the paper. XH and JL performed all statistical analyses and contributed to revising the paper. XX helped to find the source and analysis method. XHH helped to revise the work critically for important intellectual content, approved the final version to be published, and agreed to be accountable for all aspects of the work. All authors have read and approved the manuscript.
